# The Impact of a Flexible Stern on Canoe Boat Maneuverability and Speed

**DOI:** 10.3390/biomimetics5010007

**Published:** 2020-02-17

**Authors:** Anna Theresia Stadler, Martin Schönauer, Roozbeh Aslani, Werner Baumgartner, Tillmann Philippi

**Affiliations:** 1 Institute of Biomedical Mechatronics, Johannes Kepler University Linz, Altenberger Str. 69, 4040 Linz, Austria; martin.adastra@gmail.com (M.S.); werner.baumgartner@jku.at (W.B.); tillmann.philippi@liwest.at (T.P.); 2 Institute of Fluid Mechanics and Heat Transfer, Johannes Kepler University Linz, Altenberger Str. 69, 4040 Linz, Austria; roozbeh.aslani@jku.at

**Keywords:** flexible stern, Fin Ray Effect^®^, canoe boat, lateral boat movement

## Abstract

Paddle boats like canoes and kayaks draw a sinusoidal path when a linear movement is intended. The reason for this behavior is that each paddle stroke induces a lateral movement of the boat. In this study, we sought to reduce the so-called yawing motion. We therefore replaced the stiff stern by a flexible stern, which is based on the Fin Ray Effect^®^. We built down-scaled boat models and tested them in a water channel. The similarities between experimental and original setup were evaluated by means of a dimensional analysis. (Thermoplastic) elastomers with various flexibility were used for the stern construction. In the experiments conducted in the water channel, we determined the forces acting on the boat with different stern models. The results reveal that the flexible stern induced a torque counteracting the boat’s deflection, while the stiff stern caused a torque enhancing it. A paddle boat with a flexible stern could hence be a promising new method to reduce the boat’s yawing movement.

## 1. Introduction

The goal in racing sports is always to enhance speed. Various methods are used to reach higher velocities. In canoe sports, including both canoe and kayak boats (The International Canoe Federation (ICF) defines each discipline and the boats used in it in detail. Canoe and Kayak boats are defined as follows: “In a canoe, the paddle has a single-blade and the athlete uses a striding position with one knee on the deck and the other foot forward allowing room to pull the paddle down their preferred side of the canoe. In a kayak, the paddler is seated and uses a double-bladed paddle pulling the blade through the water on alternate sides to propel the boat forward.”) [1], the shape and material of the boat and the paddle are the most important factors for gaining speed and improving maneuverability. In the 1980s, it was shown that the shape of the paddle had a huge impact on boat speed [2]. Caplan revealed in his study [3] that the correct paddle orientation during each stroke has a significant impact on boat velocity. In addition, drag is an issue; in order to reduce it, different coatings were tested. Most of the materials used, however, are either toxic or highly persistent; the long- and short-term effects on the aquatic ecosystem are fatal [4]. A more promising approach to reduce drag is surface structuring. The shark skin is one famous structure and seems to be a promising alternative [5,6]. The Speedo^®^ shark-skin swimsuit is certainly the most successful example; in the 2000 Summer Olympics in Sydney, 83% of medals were won by swimmers using this suit.

In this work, we contemplated the problem from another point of view: We were keen to investigate the causes of energy losses in canoe movements. The streamline shape and the material of the boat should guarantee smooth gliding, i.e., minimize drag. The shape of the paddle should optimally transmit the force of the paddler into the water. However, each paddle stroke inevitably induces a yaw due to its off-center point of force, which needs to be corrected, and the boat draws a sinusoidal path while moving through water. The possibility to reduce the amplitudes of this path and thereby making rowing more efficient was studied.

The plan was to use a biomimetic top-down approach: (1) to define the problem (yawing effect), (2) search for analogies in biology, (3) identify a principle and abstract it, and finally, (4) test and analyze the abstracted model.

**Figure 1:**
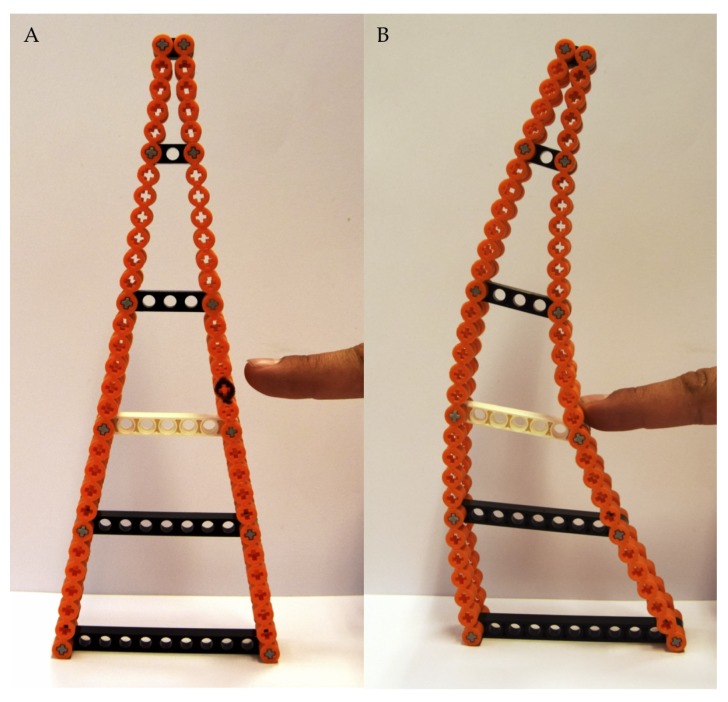
(**A**) shows the characteristic Fin Ray geometry, and (**B**) the Fin Ray Effect^®^.

During phase (2), we discovered an interesting biomimetic abstraction, the so-called Fin Ray Effect^®^: In 1997, during fishing holidays in Norway, Leif Kniese saw that fish fins bent around his finger when he applied pressure instead of bending away [7]. The Fin Ray Effect^®^ (Evologics GmbH, Berlin, Germany) was discovered [8]. The principle of this effect is based on a characteristic triangular geometry consisting of a rigid skeleton (cross braces) and flexible side walls (Figure 1). The most famous application is the Fin Ray gripper. It can handle delicate objects, easily conforms to an object, and can be applied for robots with human contact [9,10].

We decided to use this abstracted model and adapt it for our problem, i.e., to design a flexible boat stern. The idea of a flexible boat is not new. When scientists explored the Arctic regions, they were fascinated by the Inuit boats because of the high speed and maneuverability. They studied the boats, and discovered that the Inuit used flexible materials and that the boats therefore experienced less drag forces—they smoothly glided through water. These so-called baidarka kayak boats were used by the natives of the Aleutian Islands for water transportation and hunting. Since trees and wood were scarce, the Aleutian people relied on driftwood and bones to create the frame, which was covered with the skins of sea animals. With their rigid core and flexible skin, these boats were highly resistant against the harsh environments they were used in [11].

Here, we sought to bring the Fin Ray Effect^®^ back into water. To reduce the yawing effect described above, a boat with a flexible stern was designed. The stern is based on the Fin Ray Effect^®^, meaning that it moves towards the force induced by the paddle. To study the impact on boat speed, down-scaled boat models were designed and tested in a water channel. For the construction of the stern, elastomers with various flexibility (ranging from stiff to highly flexible) were used. We measured the lateral force that would lead to a yawing motion on different boat models and compared the results. They show that the flexible stern damps the lateral boat movement. We believe that it is a promising method to enhance canoe boat speed.

The primary objectives of this work were to (a) to prove that a flexible stern based on the Fin Ray Effect^®^ damps the boat’s yawing, and (b) to design a boat model based on an original canoe boat and test its applicability towards design, material, and flexibility.

## 2. Materials and Methods

### 2.1. Dimension Analysis

The dimension analysis provides a way to carry out experiments with a down-scaled model and make statements about the behavior of an upscaled model. To accomplish this, the problem must be described dimensionless; therefore, all relevant parameters must be related to characteristic parameters. This method assures that the results are not only relevant for the studied model but also for all similar models. In this case, similarity means that all dimensionless parameters have the same value.

To determine these parameters, we used the Buckingham pi theorem. The following ten variables were defined for the flexible stern problem: periodic duration of the oscillation (T in s), relative velocity between water and boat (*v* in $\frac{m}{s}$), density of the medium (ρ in $\frac{\mathrm{kg}}{m^{3}}$, $\rho_{w}$ = 997 $\frac{\mathrm{kg}}{m^{3}}$), kinematic viscosity of the medium (ν in $\frac{m^{2}}{s}$, $\nu_{w}$ $=10\;\times \;10^{-6}$ $\frac{m^{2}}{s}$ at a temperature of $20^{\circ }C$), cross-section area of the boat in water (*A* in $m^{2}$), length of the boat (*l* in m), gravitational acceleration (*g* in $\frac{m}{s^{2}}$, *g* = 9.81 $\frac{m}{s^{2}}$), Young’s modulus of the Fin Ray^®^ material (*Y* in $\frac{\mathrm{kg}}{m\;\cdot \;s^{2}}$), length of the flexible boat stern ( $l_{r}$ in m), and wall thickness of the boat stern model ($d_{wt}$ in m). Using the above variables, the problem could be characterized by the following dimensionless parameters (Table 1):

The Strouhal number is the dimensionless frequency of the vortex shedding. The Reynolds number is defined as the ratio of the inertial forces to the viscous forces [12]. The Froude number is defined as the ratio between inertial force and gravitational force. If the Froude number is similar in both models, then the wave system is similar [12]. The Cauchy number is the ratio between inertial and elastic forces [13] and is used in compressible fluids or with objects made from an elastic material. The other three are geometric ratios.

### 2.2. Models

**Figure 2:**
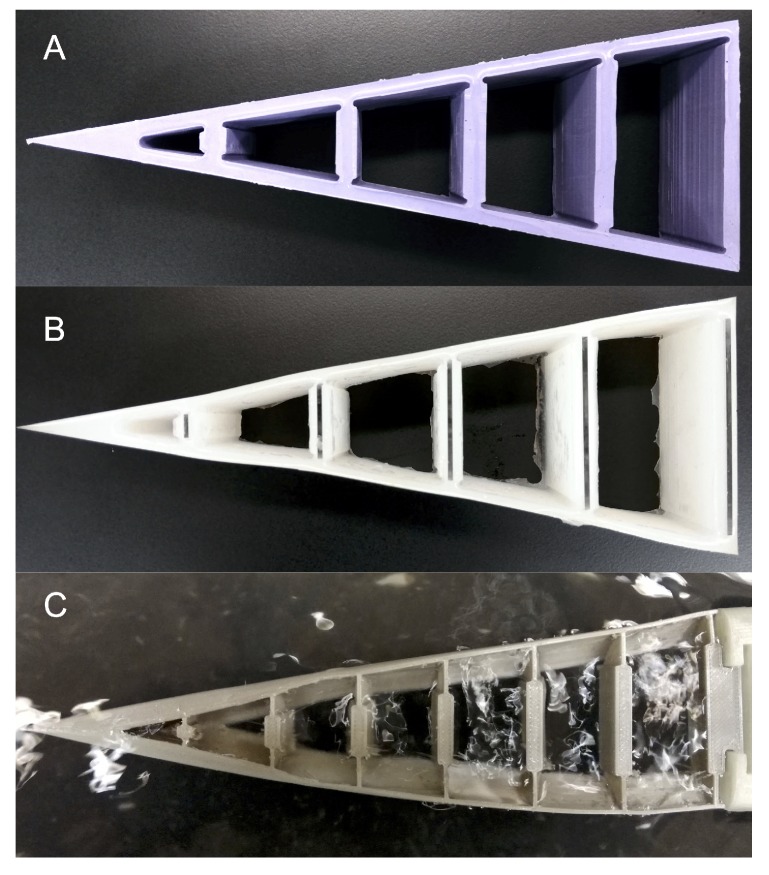
Stern models (**A**–**C**). Models (**A**) and (**B**) were cast from silicone rubber with different Young’s modulus to proof our hypothesis. Model C was 3D-printed from thermoplastic polyurethane (TPU, NinjaFlex^®^, NinjaTek^®^, Manheim, PA, USA) and represents the stern of a down-scaled canoe boat model.

Generally, three different models were used to investigate the effect of the Fin Ray on the lateral forces acting on the boat (Figure 2). For a principal estimation, simple stern models (A and B) were used. At the beginning, these models were cast with a thin bottom membrane. The membrane however reduces the flexibility of the side walls and hence eliminates the Fin Ray Effect^®^. Therefore, the models were designed without a bottom membrane. After successful testing of the simple Fin Ray geometries (A and B), we designed the down-scaled boat model C based on an original canoe boat.

#### 2.2.1. Stern Models A and B

**Figure 3:**
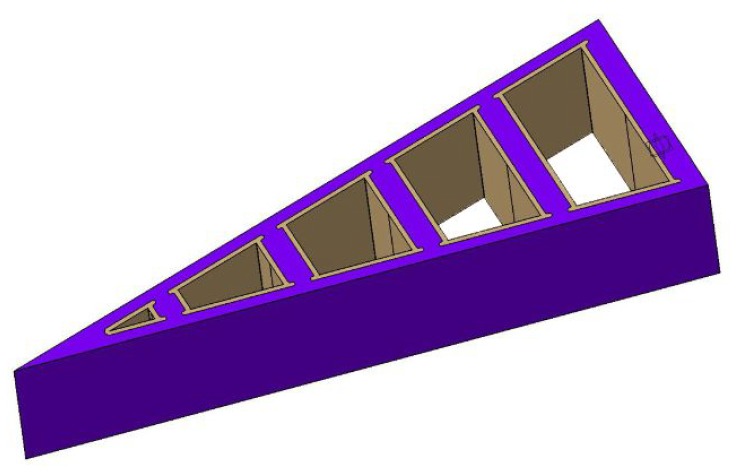
The draft for stern models A and B (in violet) with braces (in beige) to create the stiff configuration of the stern models.

Stern models A and B were made from silicone rubber. For model A, Smooth-Sil^®^ 945 with a Young’s modulus of $Y_{A}$ $=1.79\;\times \;10^{6}$ $\frac{\mathrm{kg}}{m\;\cdot \;s^{2}}$, and for model B Ecoflex^TM^ 00-30 (both Smooth-on, Macungie, PA, USA) with a Young’s Modulus, $Y_{B}$ $=6.895\;\times \;10^{4}$ $\frac{\mathrm{kg}}{m\;\cdot \;s^{2}}$ was used. Both models were cast with a mold to obtain the characteristic Fin Ray shape. Since the silicone rubber used in model B is highly flexible, the cross braces were shored up with Poly(methyl methacrylate) (PMMA) elements to retain the Fin Ray effect. We conducted alternating experiments, where we measured the forces acting on both the stiff and the flexible model. To guarantee the same measurement conditions between the experiments, the stiff models were designed by adding stiff braces (Figure 3) to the flexible model. These braces were held in place by friction and prevented any flexion of the stern, thereby creating a stiff reference model. The boat hull is a down-scaled canoe boat model (scale factor 1:5).

The total length of both boat models (hull and stern) was $l_{AB}$ =1 m, whereas the boat hull was 0.73 m and the boat stern $l_{rAB}$ =0.27 m long. The maximum width of the the models was 0.11 m. Due to the usage of 3D-printed and laser cut molds, we could achieve solid tolerances of ±2%.

#### 2.2.2. Stern Model C

**Figure 4:**
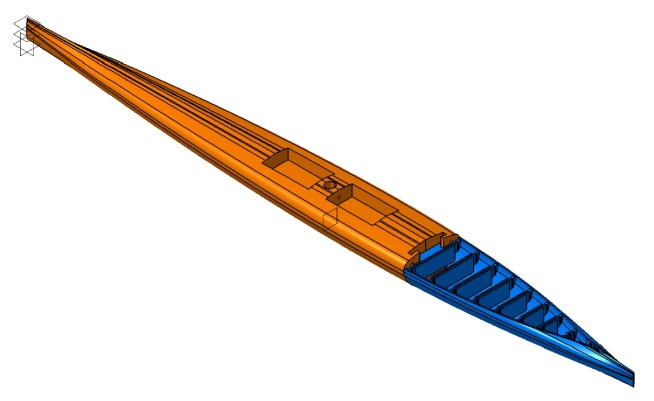
The draft for model C with hull in orange and stern in blue and the interlocking system for a convenient connection.

The flexible version of stern model C was 3D-printed from thermoplastic polyurethane (TPU, NinjaFlex^®^, NinjaTek^®^, Manheim, PA, USA) with a Young’s modulus of $Y_{C}$ $=1.242\times 10^{7}$ $\frac{\mathrm{kg}}{m\;\cdot \;s^{2}}$ [14]. The stiff version was printed from polylactic acid (PLA), a stiff bio-polymer. The front of the boat was also printed from PLA (Figure 4). While we were testing models A and B, it was shown that even a thin skin at the bottom eliminates the Fin Ray Effect^®^. Therefore, model C was designed bottomless to guarantee a reduction of the yawing.

With a length of $l_{C}$ =0.5 m, a stern length of $l_{rC}$ =0.135 m and a width of 0.055 m model C was only half the size of A and B. The reason for the down-scaling was that we could still print the entire boat model (hull and stern) on a standard 3D-printer.

### 2.3. Experimental Setup

From a kayak’s point of view, the water is moving towards it. The yawing motion temporarily shifts the water velocity from directly frontal to slightly lateral at an angle. This angle puts lateral water pressure on the flexible stern, which bends towards the pressure due to the Fin Ray effect, and thus applies a force in the direction opposite to the yawing.

**Figure 5:**
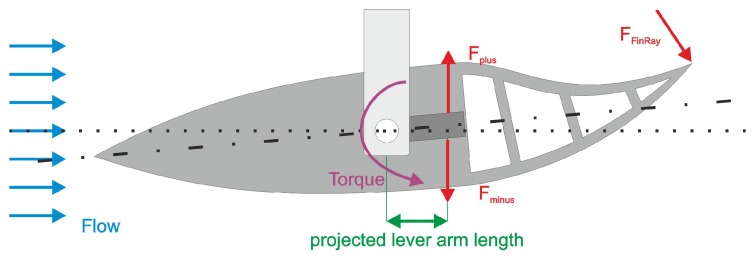
Measurement setup. The boat was deflected by $5^{\circ }$ to measure the forces $F_{plus}$ and $F_{minus}$ by means of two strain gauge measurement bridges.

The same situation could be achieved in a water flow channel by mountig the model at its center point with a rod supported by ball bearings (Figure 5) deflecting it by $5^{\circ }$. The maximum flow velocity was $v_{c}$ =0.28 $\frac{m}{s}$. Two strain gauge measurement bridges (Tedea-Huntleigh 1004, Vishay Loadcells Intertechnology Inc., Toronto, Ontario, Canada) were needed to measure both positive and negative magnitudes. Strain gauge (a) was used for the positive values ($F_{plus}$), which showed how much the boat kept rotating, and strain gauge was measuring the negative values ($F_{minus}$), which indicate how much the boat counteracted the deflection. The measurement results of both sensors were multiplied by their respective effective lever arm lengths to obtain the torque values ($M_{plus}$ and $M_{minus}$) acting on the model.

To prevent lost motion, a slight preloading had to be applied to the sensors. This preload was measured prior to each measurement cycle with the flow channel turned off, $v_{preload}$ =0.00 $\frac{m}{s}$, and subsequently subtracted from the measurement data. Three experiments were conducted to test the three stern models A to C in both their flexible and stiff configurations. Each experiment consisted of ten measurements, alternating between the flexible and stiff configuration. Each measurement lasted 30 s; 3000 measurement points were collected with Matlab (R2015a, MathWorks, Natick, MA, USA).

$M_{minus}$ was multiplied by −1 because the strain gauge measures the movement in the opposite direction of $M_{plus}$. Finally, the two torques $M_{minus}$ and $M_{plus}$ were summed up to get the total torque *M*.

## 3. Results and Discussion

To prove that a flexible boat stern based on the Fin Ray Effect^®^ reduces the paddle-stroke-induced yawing, we designed and manufactured a simple geometry (models A and B) reflecting the classic Fin Ray gripper [15]. Then, a down-scaled boat model with a flexible, Fin Ray-stern was drafted based on an original canoe boat to verify that only a slight adaptation of a standard boat can have a significant impact on the yawing movement.

### 3.1. Dimension Analysis

Due to their definitions, it is impossible to achieve Reynolds and Froude similarity at the same time. With the available equipment, we could achieve neither Reynolds nor Froude similarity because the maximum flow velocity in the water channel was limited to $v=0.28\frac{m}{s}$. Professional canoeists, however, reach velocities between approximately 2 and $6\frac{m}{s}$ [16].

Since the characteristic time was defined as the time that one water molecule needs to pass the boat ($T=\frac{l}{v}$ ), the Strouhal number was normalized to the value 1 for now. When using the time between paddle strokes in a real size experiment, the Strouhal number can gain more relevance. The Cauchy number varies between the models, since materials with different Young’s Moduli were tested. The geometric dimensionless quantities are similar for models A and B, as the wall thickness, the cross-sectional area of the boat that is in water, and the total length were adapted accordingly. Model C varies due to its different geometric structure and production method. All parameters are displayed in Table 2.

This makes it difficult to draw assumptions for the implementation in an original boat. Therefore, real-scale experiments have to be done to prove the applicability of our results for larger models, since a simple upscaling based on the dimension analysis is not feasible. The evidence that the deflecting torque is reduced in all experiments compared to a stiff model indicates that the principle of a Fin-Ray-Effect^®^-based, flexible stern could reduce the boat’s yawing motion and hence enhance maneuverability and speed.

### 3.2. Experimental Results

**Figure 6:**
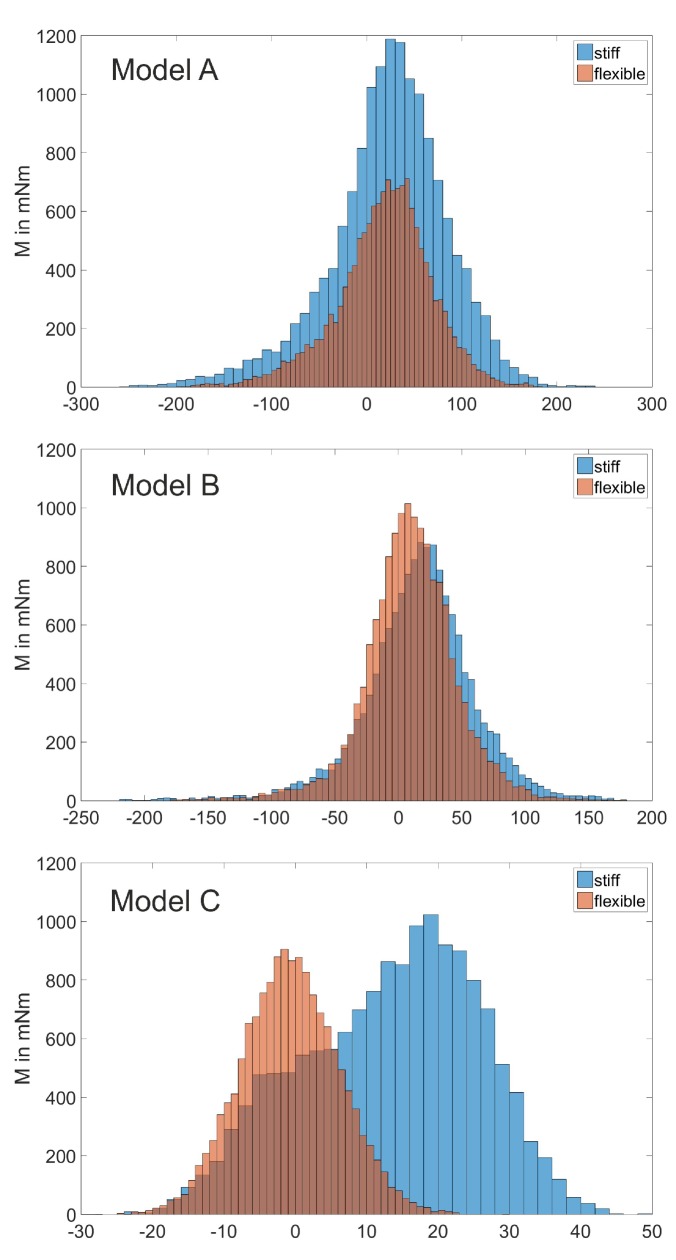
Histograms of the total torque of the three stern models and their stiff counterparts. While in model A the mean value is not reduced by much, the torque’s overall amplitude is reduced significantly. In model B, the mean shifts to lower values, but the total amplitudes are increased. This is caused by the very flexible model, resulting in high vibrations. In model C, the shift to lower torque values, and therefore less yaw, is significant. The amplitude of the oscillations is also slightly reduced.

Figure 6 displays the results of the three experiments. In all three experiments, the total torque was lower in the flexible stern than in the stiff counterpart; when looking at the measurement data of model C, the difference between the total torque of the flexible and the stiff model becomes more obvious because the range of the total torque (namely between −30 and 50 mNm) is lower and hence resolution is higher than in the data sets of models A and B (−250 to 250 mNm). The mean values including the standard deviation of the data sets are introduced in Table 3. They emphasize the tendencies shown in Figure 6, as all the mean values of the boat models with a flexible stern are lower than those for the stiff stern.

This means that in all three experiments the model with the flexible stern experiences less torque in the direction of the deflection and therefore reduces the yawing effect. In model C, the down-scaled boat model, the flexible stern even counteracts the deflection as the total torque results in a slightly negative value of −0.72 mNm.

The wide variation of the total torque in models A and B may be explained by (a) the inaccuracy of the setup because hull and stern were connected with fabric tape; and (b) the higher flexibility of silicone rubber (in comparison to TPU), which can lead to higher vibrations. The stiff stern models consisted of the flexible stern with stiff elements as braces (see Figure 4). With this practical solution, the setup had to not be changed between the measurements and therefore inaccuracies were avoided.

In model C, an interlocking system connected hull and stern. The switching between stiff and flexible stern models between the measurements was simple and fast. Although this flexible stern model had a higher Young’s modulus than models A and B (see Section 2.2), and an additional loss of flexibility due to the addition of curvature to the Fin Ray cross-section (because the model’s geometry was adapted to the geometry of the original canoe boat), model C even counteracted the deflection movement. We tried to compensate the aforementioned by reducing the stern’s wall thickness to a minimum and by designing a bottomless model.

## 4. Conclusions

In this work, we sought to reduce the boat’s yawing movement induced by paddle strokes. We demonstrated that a flexible stern counteracts a boat model’s lateral movement, when it is deflected. These results indicate that a canoe boat with a flexible stern based on the Fin Ray Effect^®^ would experience less yawing due to its deflection movement.

We started experimenting with a down-scaled boat model in order to demonstrate this hypothesis. The future goal is to adapt an original canoe boat with a flexible stern, based on our model C which performed best in our experiments. We want to test both a flexible boat and a stiff model in a water channel to compare the paddle-stroke-induced yawing effects.

## Figures and Tables

**Table 1:** Four known and three geometric dimensionless quantities. Str denotes the Strouhal number, Re the Reynolds number, Fr the Froude number, and Ca is the Cauchy number.

$\pi_{1}=Str=\frac{l}{T\;\cdot \;v}$	$\pi_{2}=Re=\frac{l\;\cdot \;v}{\nu }$	$\pi_{3}=\frac{A}{l^{2}}$	$\pi_{4}=Fr=\frac{v^{2}}{l\;\cdot \;g}$
$\pi_{5}=Ca=\frac{v^{2}\;\cdot \;\rho }{Y}$	$\pi_{6}=\frac{l_{r}}{l}$	$\pi_{7}=\frac{d_{wt}}{l}$	

**Table 2:** Dimensionless parameters.

Model	Re	Str	Fr	$\Pi_{3}$	Ca	$\Pi_{6}$	$\Pi_{7}$
A	$2.8\;\times \;10^{5}$	1	$7.99\;\times \;10^{-3}$	$5.50\;\times \;10^{-3}$	$4.36\;\times \;10^{-5}$	0.27	$1\;\times \;10^{-2}$
B	$2.8\;\times \;10^{5}$	1	$7.99\;\times \;10^{-3}$	$5.50\;\times \;10^{-3}$	$1.13\;\times \;10^{-3}$	0.27	$1\;\times \;10^{-2}$
C	$1.4\;\times \;10^{5}$	1	$1.60\;\times \;10^{-2}$	$2.00\;\times \;10^{-2}$	$6.29\;\times \;10^{-6}$	0.27	$2\;\times \;10^{-3}$

**Table 3:** Experimental results, showing the mean values ± standard deviation of the total torque *M* for the stiff and the flexible stern models.

	Model A	Model B	Model C
$M_{stiff}$ (mNm)	22.76±19.04	16.54±3.96	13.27±11.70
$M_{flex}$ (mNm)	18.62±14.70	9.44±6.27	−0.72±2.54

## Abbreviations

The following abbreviations are used in this manuscript:

ICF	International Canoe Federation
PMMA	Poly(methyl methacrylate)
Str	Strouhal number
Re	Reynolds number
Fr	Froude number
Ca	Cauchy number
TPU	Thermoplastic urethane
PLA	Polylactic acid
